# Structural and Ultrastructural Analysis of Cerebral Cortex, Cerebellum, and Hypothalamus from Diabetic Rats

**DOI:** 10.1155/2009/329632

**Published:** 2009-10-01

**Authors:** Juan P. Hernández-Fonseca, Jaimar Rincón, Adriana Pedreañez, Ninoska Viera, José L. Arcaya, Edgardo Carrizo, Jesús Mosquera

**Affiliations:** ^1^Instituto de Investigaciones Clínicas Dr. Américo Negrette, Facultad de Medicina, Universidad del Zulia, Maracaibo 4001-A, Zulia, Venezuela; ^2^Cátedra de Inmunologia, Escuela de Bioanalisis, Facultad de Medicina, Maracaibo 4001-A, Zulia, Venezuela; ^3^Instituto de Investigaciones Odontológicas, Facultad de Odontología, Universidad del Zulia, Maracaibo 4001-A, Zulia, Venezuela

## Abstract

Autonomic and peripheral neuropathies are well-described complications in diabetes. Diabetes mellitus is also associated to central nervous system damage. This little-known complication is characterized by impairment of brain functions and electrophysiological changes associated with neurochemical and structural abnormalities. The purpose of this study was to investigate brain structural and ultrastructural changes in rats with streptozotocin-induced diabetes. Cerebral cortex, hypothalamus, and cerebellum were obtained from controls and 8 weeks diabetic rats. Light and electron microscope studies showed degenerative changes of neurons and glia, perivascular and mitochondrial swelling, disarrangement of myelin sheath, increased area of myelinated axons, presynaptic vesicle dispersion in swollen axonal boutoms, fragmentation of neurofilaments, and oligodendrocyte abnormalities. In addition, depressive mood was observed in diabetic animals. The brain morphological alterations observed in diabetic animals could be related to brain pathologic process leading to abnormal function, cellular death, and depressive behavioral.

## 1. Introduction

Besides autonomic and peripheral neuropathy, diabetes is also associated with gradually developing end-organ damage in the central nervous system [1]. This complication is referred as “diabetic encephalopathy” and characterized by impairment of cognitive functions and electrophysiological changes [2]. These functional changes are accompanied by structural and neurochemical abnormalities as well as by degenerative changes in the brain that could be related to chronically increased intracellular glucose concentration [3–5]. Several brain regions have been studied by biochemical and structural analysis in diabetes; however, few studies have been performed to determine the ultrastructural features of diabetes in cerebral cortex, hypothalamus, and cerebellum. These regions are involved in cognitive, motor, and neuroendocrine activities [6–9]; thus, their affectations during diabetes are relevant in the pathogenesis of the disease. This study was aimed to characterize the structural and ultrastructural brain changes in streptozotocin (STZ) induced diabetic rats.

## 2. Methods

### 2.1. Streptozotocin-Induced Diabetes

Male Sprague-Dawley rats weighing 100–150 g (Instituto Venezolano de Investigaciones Cientificas, IVIC) were used. Animals were housed, and food and water were provided ad libitum. Diabetes was induced by intravenous injection of streptozotocin (55 mg/kg in 0.2 M sodium citrate, pH 4.0; Sigma-Aldrich, St. Louis, MO, USA). Control rats (*n* = 10) were injected with vehicle. Diabetes was verified at weeks 1, 6, and 8 by measurement of tail blood glucose levels using a glucometer (Lifescan, Milpitas, CA, USA). Rats having a blood glucose level of ≥200 mg/dL were considered to be diabetic (*n* = 8). To prevent hypoglycemia, rats were allowed unlimited access to water with 2% glucose during 3 days after STZ injection. Subsequently, rats were allowed unlimited access to tap water. Urine glucose and ketoacids (assessed as acetoacetate) were evaluated using Multistix urinalysis strips (Bayer; Fisher Scientific, Germany). After 8 weeks, control and hyperglycemic rats were anesthetized by an intraperitoneal injection of tiobarbituric, and brain was perfused by intracardiac via using a 0.9% of saline solution containing 4% of glutaraldehyde. Thereafter, cerebrum and cerebellum were obtained for microscopy studies. Experimental procedures followed the ethical guidelines of the committee of bioethical and biosecurity of FONACIT (Caracas, Venezuela) and the committee of bioethical of Medical School (Universidad del Zulia, Maracaibo, Venezuela).

### 2.2. Light and Electron Microscopy

Pieces of selected brain regions (cerebral cortex, hypothalamus and cerebellum) were immediately placed in 2.5% glutaraldehyde in 0.1 M cacodylate buffer, chopped to get pieces of approximately 1 mm^2^, and kept in the same glutaraldehyde solution for 12 hours at room temperature. Samples were postfixed in 1% osmium tetroxide for 1.5 hours, dehydrated in increasing concentrations of alcohol, immersed in propylene oxide, and embedded in Araldite 502 resin at 60°C. To confirm that hypothalamic area and all layers of cerebral and cerebellar samples were analyzed, thick tissue sections were made, stained with toluidine blue and analyzed by light microscopy. Ultrathin sections were placed on grids and stained with uranyl acetate and lead citrate. The thick sections were examined using a light microscope (Axioscop, Zeiss, Germany), and quantitative analysis of myelinated axonal transversal areas (cytoplasm areas) was performed using an IA32 system software (LECO Corporation, MI, USA); the grids with thin sections were examined in a transmission electron microscope (JEOL 1010, Akishima, Japan).

### 2.3. Quantification of Myelin and Mitochondrial Alterations

**Figure 1:**
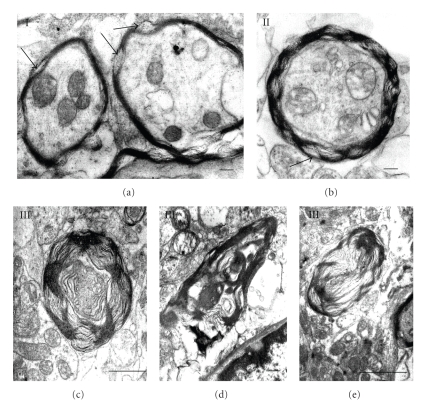
Grades of myelin alterations (I, II, and III). Arrows indicate local disarrangement of myelin sheath. Scale bars (a) and (b): 0.2 *μ*; (c): 1 *μ*; (d): 0.5 *μ*; (e): 2 *μ*. There are uranyl acetate and lead citrate staining.

**Figure 2:**
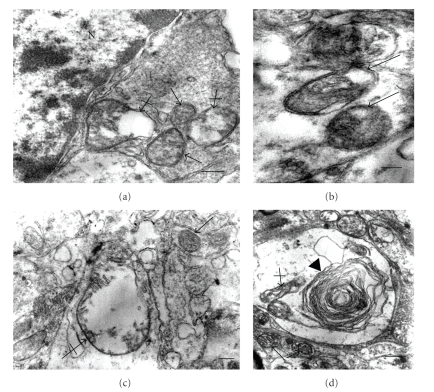
Mitochondrial alterations in diabetes. (a) Normal (arrows) and swollen mitochondria (crossed arrows) in neuronal body. (b) Vacuolated mitochondria in neuropil (arrows). There is intense mitochondrial swelling in neuropil (crossed arrow); note a normal mitochondria (arrow). (d) Contribution of mitochondrial degeneration (crossed arrow) to myelin like formation (arrow head); note two normal mitochondria (arrows). Bars in (a), (b), and (c) 0.2 *μ*; (d): 0.5 *μ*. There are uranyl acetate and lead citrate staining.

Randomly selected myelinated axons (100 per each cerebral cortex, hypothalamus or cerebellum areas) were counted and classified using an electron microscope. Abnormal myelin was arbitrarily defined on the basis of myelin disarrangement in type I: well-preserved myelin with local disarrangement of myelin sheath, type II: diffused local disarrangement of myelin sheath resembling a “collar” but preserving structural arrangement, and type III: collapsed myelin to form ovoid, diffused disarrangement, fusion or broken myelin sheath (Figure 1). Mitochondrial alterations were studied in randomly selected cellular body and neuropil areas (100 per each area) using an electron microscope. These organelles were counted and classified as vacuolated mitochondria (VM) and swollen mitochondria (SM) (Figure 2).

### 2.4. Apoptosis

To determine apoptosis a histological assay for selective labeling of cells with degraded DNA in tissue sections was used [10]. Apoptosis was determined using a commercial kit following the manufacturer's indications (Promega, Madison, WI, USA). Areas of randomly selected tissues were analyzed and results were expressed as number of apoptotic nuclei per 0.065 mm^2^ of area. Apoptotic morphological features were also study by electron microscopy procedures.

### 2.5. Forced Swimming Test (FST) and Motor Activity

This test was performed with modifications of the Porsolt's procedure [11] to determine the struggling time in control and diabetic rats. Briefly, FST was performed as follows: 8-week diabetic and control rats were placed individually in glass cylindrical tanks containing water (25°C) at a depth of 30 cm. FST was performed daily for 15 days, and the struggling time was measured during 30-minute test. A rat was judged to be immobile when it remained floating in the water and made only small movements to keep its head above water. Struggle occurred when the rats were diving, jumping, strongly moving all four limbs, or scratching the walls. Animals were tested for motor activity in an optical digital animal activity monitor (Opto-Varimex-Minor, Columbus Instruments Co., Columbus OH, USA), which records motor activity values (pulses) automatically. Prior to the FST, the animals were placed for 5 minutes in the activity monitor where total horizontal activity and ambulatory and stereotypic movements were obtained.

### 2.6. Statistical Analysis

Data are expressed as mean ± SD. Statistical significance was determined by unpaired *t*-test using Graph Pad InStat. Differences with *P* < .05 values were considered statistically significant.

## 3. Results

### 3.1. Biochemical Parameters

STZ injection produced diabetic animals with consistent high levels of plasma glucose (Week 1: 409.13 ± 92.55; Week 6: 363 ± 77.92; Week 8: 306.4 ± 84.66 mg/dL). Table 1 shows the biochemical parameters at week 8 when animals were sacrificed. Increased plasma glucose and urine glucose excretion were observed. At this time urine ketoacids were not detected. Decreased weight gain was observed in diabetic animal when compared to controls.

### 3.2. Light Microscopy Analysis

**Figure 3:**
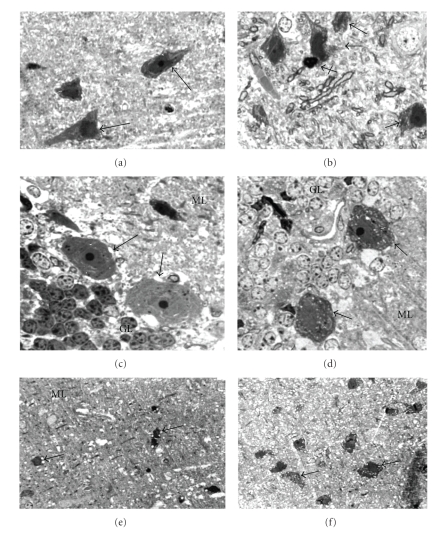
Structural alterations in diabetes. (a) Normal cerebral pyramidal cells (arrows). (b) Vacuolization of pyramidal cells in diabetes (arrows) and a probably apoptotic cell (crossed arrow). (c) Purkinje cells (arrows) in normal cerebellar section. (d) Vacuolization of Purkinje cells (arrows); note also vacuolization of granular layer cells. (e) Vacuolization of neuropil and cells (arrows) in cerebellar molecular layer. (f) Vacuolization of dark cells (arrows) and neuropil in hypothalamus of diabetic rat. GL: granular layer; ML: molecular layer. Magnification: ×1000 (a)–(d); ×400 ((e) and (f)). There is toluidine blue staining.

**Figure 4:**
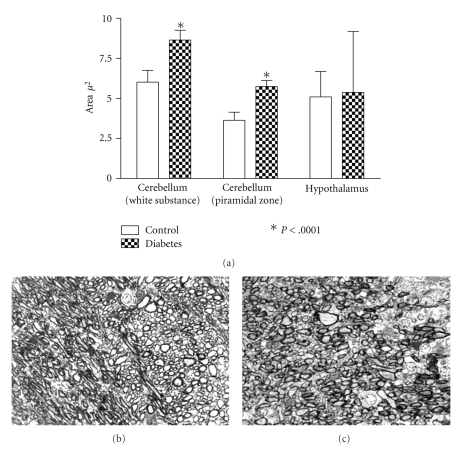
Area of mielinated axons in diabetes. (a) Axonal area of myelinated axons. (b) Normal cerebellar area (white substance) showing myelinated axons. (c) Diabetic cerebellar area (white substance) showing myelinated axons. Magnification: ×400 ((b) and (c)). There is toluidine blue staining.

Analysis of toluidine-stained sections showed vacuolization of neurons, glia, as well as the neuropil in cerebral cortex, hypothalamus, and cerebellum of diabetic rats. Cellular vacuoles were mainly observed in pyramidal cells, Purkinje cells, and cells of cerebellar granulose and molecular layers. Cells with picnotic nuclei and condensed cytoplasm suggesting apoptotic cells were observed in normal and diabetic animals (Figure 3). Analysis of transversal sections of myelinated axons showed increased area in cerebral and cerebellar axons when compared to normal (Figure 4).

### 3.3. Apoptosis

**Figure 5:**
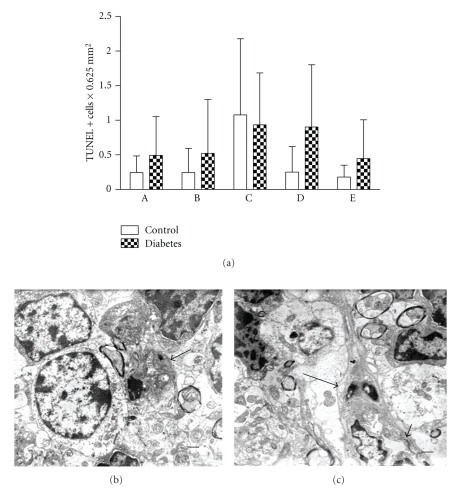
Apoptosis in central nervous system of normal and diabetic rat. (a) TUNEL positive cells: A: Cerebral molecular-granulose zone. B: Cerebral pyramidal zone. C: Choroids plexus. D: Cerebellar granulose zone. E: Cerebellar molecular zone. (b) Apoptotic cell in cerebellum (arrow). (c) Perivascular apoptotic cell in cerebellum (arrow). Short arrow indicates capillary. Scale bars are 1 *μ*. There are uranyl acetate and lead citrate staining.

Presence of apoptosis in light microscopy analysis was confirmed by TUNEL assay and by electron microscopy. Increased numbers of TUNEL positive cells were observed in cerebral cortex and cerebellum in diabetic animals when compared to controls, but they failed to reach a statistical significance. Electron microscopy study showed cells with condensed chromatin and cytoplasm in the brain parenchyma; some of these cells represent endothelial and perivascular cells (Figure 5).

### 3.4. Ultrastructural Analysis

**Figure 6:**
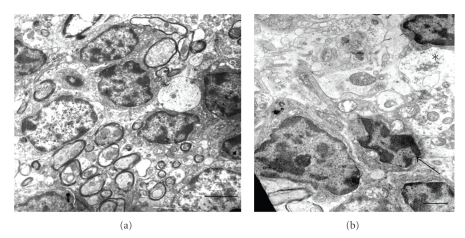
Normal and diabetic cerebellum. (a) Normal granulose zone; Bar: 2 *μ*. (b) Diabetic granulose zone showing cell (arrow) with swollen mitochondria. Asterisk indicates swollen neuropil; Bar: 1 *μ*. There are uranyl acetate and lead citrate staining.

**Figure 7:**
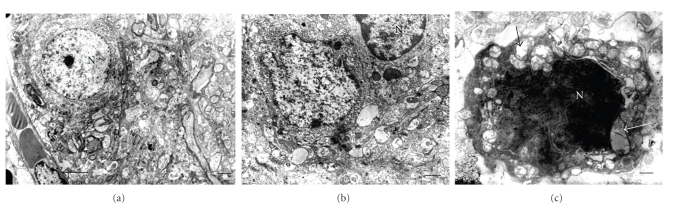
Normal and diabetic cerebral cortex. (a) Normal cerebral area. Arrow indicates capillary; Bar: 2 *μ*. (b) Diabetic cerebral zone showing swollen neuropil; Bar: 1 *μ*. (c) Dark cell showing different grades of mitochondrial swelling (arrows); Bar: 0.5 *μ*; N: nucleus. There are uranyl acetate and lead citrate staining.

**Figure 8:**
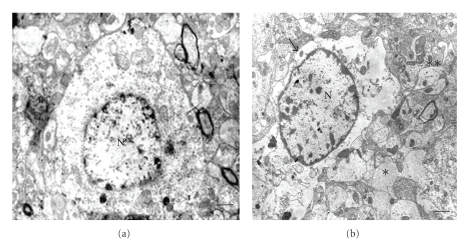
Normal and diabetic hypothalamus. (a) Normal hypothalamus area; Bar: 2 *μ*; (b) Diabetic hypothalamus area showing a swollen (asterisk) and normal (double asterisk) neuropil areas. Arrow indicates swollen cell; N: nucleus; Bars: 1 *μ*; There are uranyl acetate and lead citrate staining.

Cerebral cortex, hypothalamus, and cerebellum showed ultrastructural features suggesting general swelling of neurons, glia, and neuropil. Cellular swelling was represented by local cytoplasm swelling, dilated rough endothelial reticulum (RER) fragments and swollen organelles mainly mitochondria, or by total cellular swelling (Figures 6, 7, and 8).

**Figure 9:**
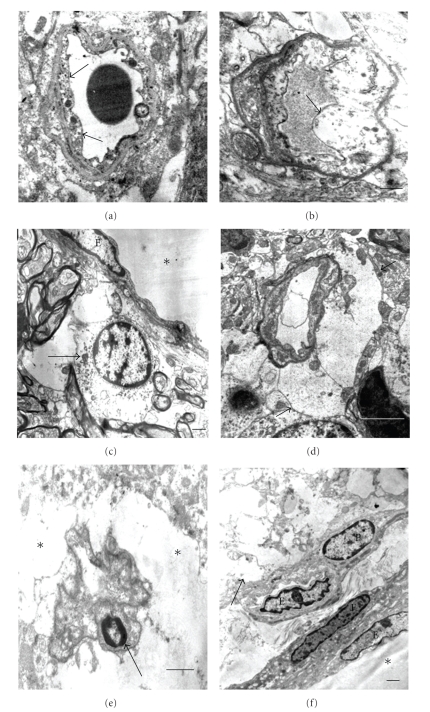
Central nervous system blood vessels in diabetes. (a) and (b) Diverse grades of endothelial cytoplasm swelling (arrows); bars: 0.5 *μ*. (c) Swelling of perivascular cell (arrow); E: endothelial cell; asterisk: vascular lumen; bar: 1 *μ*. (d) Intense perivascular swelling (arrows) preserving endothelium; bar: 2 *μ*. (e) Intense perivascular swelling (asterisks) with apoptosis of endothelial cell (arrow); bar: 1 *μ*. (f) Intense neuropil swelling (arrow) besides well-preserved blood vessels; P: pericyte; E: endothelium; F: fibroblast; asterisk: vascular lumen; bar: 1 *μ*. There are uranyl acetate and lead citrate staining.

Vascular alterations were represented by endothelial cells showing local swelling areas or intense cellular swelling. In some instance, endothelial cell apoptosis was observed (Figure 9). Frequently, intense swelling of perivascular cells was observed in several blood vessels (Figure 9). 

Mitochondrial abnormalities were observed in both cellular body and neuropil in the different regions. Increased vacuolated and swollen mitochondria in cerebral cortex, hypothalamus and cerebellum were observed in diabetic rats. No significant differences were observed when mitochondrial abnormalities in cellular body were compared with those in neuropil of diabetic animals. (Table 2 and Figure 2). Preserved mitochondria were frequently found besides altered mitochondria and, in some instance, myelin-like formations from degenerated mitochondria were observed (Figure 2).

**Figure 10:**
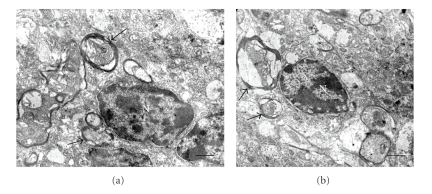
Diverse grades of oligodendrocyte abnormalities. (a) and (b) show cytoplasm and organelle swelling and altered myelinated axons (arrows); Bars: 1 *μ*. There are uranyl acetate and lead citrate staining.

**Figure 11:**
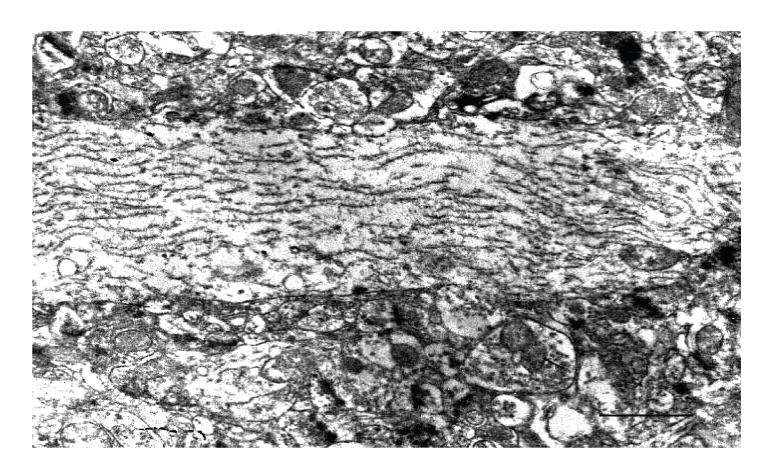
Fragmentation of neurofilaments in an unmielinated axon (arrow) in cerebral cortex; Bar: 1 *μ*. There are uranyl acetate and lead citrate staining.

**Figure 12:**
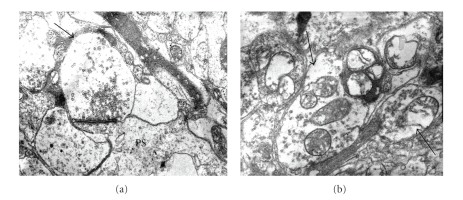
Synaptic area in diabetes. (a) Axonal bouton (arrow) showing dispersion of synaptic vesicles; PS: Postsynaptic area. (b) Swollen axonal boutons with dispersion of synaptic vesicles and mitochondrial swelling (arrows); Bars: 0.2 *μ*; There are uranyl acetate and lead citrate staining.

Axonal abnormalities were found in STZ-animals. Diabetic animals showed an elevated number of myelin alterations. Arbitrarily myelin alterations were classified as types I, II, and III according to the grade of myelin disarrangement (Figure 1). In general a low percentage of myelin alterations were observed in normal tissues. Tendency to increased myelin alterations was observed in cerebral cortex in diabetic rats, but only alteration of type III was statistically significant (Table 3). Elevated percentage of total types I, II, and III myelin alterations were observed in cerebellum and hypothalamus from diabetic animals (Table 3). Altered oligodendrocytes with local cytoplasm swelling, swollen organelles, and myelinated axons showing different types of myelin alterations were observed (Figure 10). Several both myelinated and unmyelinated axons showed fragmentation of neurofilaments (Figure 11). Synaptic boutons showed different grades of swelling with swollen mitochondria and dispersion of presynaptic vesicles (Figure 12). 

### 3.5. FST and Motor Activity

Diabetic rats showed depressive status. As shown in Table 4 diabetic animals had decreased struggle activity compared to controls when they were subjected to FST. Motor activity was also decreased in diabetes as observed in the lower number of movements and diminished ambulatory activity in diabetic animals.

## 4. Discussion

Diabetic encephalopathy, characterized by impaired cognitive functions and neurochemical and structural abnormalities, may involve direct neuronal damage. This study assesses the effect of 8-week diabetic condition on the structure and ultrastructure of relevant regions of the rat brain. In general, all regions in this study presented morphologic alterations suggesting swelling of the brain. Increased area of myelinated axons in cerebral cortex and cerebellum and diverse degree of neuronal body swelling address this observation. In this regard, brain edema is the most common serious complication of diabetic ketoacidosis in children, where mechanisms of rapid changes in serum osmolality during therapy and others such as brain ischemia have been suggested [12–15]. In these experiments, diabetic rats were not in ketoacidosis, and since they did not receive insulin, hyperglycemia could induce brain damage. Thus, hyperglycemia may cause brain acidosis and dehydration, both involved in diminished cerebral blood flow and ischemia [16–18]. Ischemia-related edema involves stimulation of brain Na-K-Cl cotransporter system facilitating edema formation and swelling of endothelial cells [19–24]. Thus, it is possible that those mechanisms contribute to the brain edema observed in this study; however, since lack of insulin may have consequences in terms of neuronal function, we cannot discard others inducers of brain alterations in diabetes. The ultrastructural lesions observed in this study are not considered to be a consequence of STZ, since a toxic effect of this compound on the central nervous system has been excluded [25].

Endothelial and perivascular edema observed in diabetic rats could induce an altered blood-brain barrier (BBB) function. Studies on animal models have shown that diabetes has deleterious effects on the BBB which may contribute to neurological complications associated to this condition [26]. Since perivascular pericytes, astrocytes, and adipocytes play an important role maintaining the function of the BBB [27, 28], perivascular swelling observed in diabetic rats could represent swollen pericytes, astrocytes, or adipocytes leading to disruption of the BBB functions. 

Mitochondrial alterations were observed as other relevant finding in diabetic rats. An arbitrary classification of vacuolated mitochondria and swollen mitochondria in order to suggest different degrees of mitochondrial lesion was used in this study. In general, the percentage of mitochondria with vacuoles preserving the mitochondrial structure was higher than swollen mitochondria, suggesting progressive mitochondrial damage. Mitochondrial structural alterations have been related to oxidative stress. Previous reports have shown swelling of mitochondria during oxidative and nitrosative stress in diabetic STZ-rats [3, 29, 30]; this alteration was prevented by antioxidant treatment [29]. This mechanism may be related to vacuolated and swollen mitochondria observed in diabetic rat brain; however, it cannot be ruled out that the oxidative stress could be a consequence of a previous mitochondrial damage by increased intracellular glucose or by the effects of other damage inducers during diabetes. As interesting observation, unaltered mitochondria were often observed beside swollen or vacuolated mitochondria suggesting a selective mitochondrial resistant to the diabetic injury or a mitochondrial compensatory effect by increasing their number. These mitochondrial alterations could be accompanied by impairment mitochondrial function. In this regard, swollen hippocampal mitochondria from 4-week STZ rats downregulate GAP-43 and MKP-1 expressions; proteins relate to memory and cognition [30]. 

Diabetic peripheral neuropathy is the most common complication of diabetes; however, brain neuropathy has not been well studied. In this study, several grades of myelin alterations were observed in the different regions of the brain. Alteration type I could degenerate to wide myelin disarrangement (type III). These myelin abnormalities may be involved in a decreased propagation of nerve impulse and contribute to the brain disorders observed in diabetes. Several mechanisms have been suggested in myelin alterations in the brain of SZT–diabetic rats, such as decreased myelin-associated glycoprotein, autoantibodies to myelin basic protein, and myelin damage induced by nitric oxide [3, 31–33].

 It is expected, in myelin abnormalities and demyelination in the central nervous system, the oligodendrocyte remyelination [34]. In this regard, ultrastructural alterations of oligodendrocytes observed in this study suggest an impairment oligodendrocyte functions. Supporting this, STZ-induced diabetes hindered both oligodendrocyte and Schwann cell remyelination; hyperglycemia inhibits Schwann cell proliferation and restricts regeneration of axons [35] and RAGE expression on oligodendrocytes during experimental diabetes induces abnormal RAGE signaling with further cellular dysfunction [36]. It is possible that similar mechanisms are acting on oligodendrocytes in the central nervous system and may be related to the oligodendrocyte ultrastructural changes observed in this study. 

Swollen axonal synaptic bouton with vacuolated and swollen mitochondria and high dispersion of synaptic vesicles was observed in the neuropil. These ultrastructural findings suggest an altered synaptic transmission and could contribute to abnormal synaptic plasticity and cognitive impairments observed in experimental diabetes [37, 38]. Dispersion of synaptic vesicles could be an early alteration, since similar findings have been reported in 9-day diabetic STZ- rats in the presynaptic hippocampal mossy fiber terminals [39]. In addition, alterations in axonal neurofilaments were also observed in this study. Several axons showed fragmentation of neurofilaments suggesting damage in the axonal transportation and impairment of neuron functions. Similarly, diabetic neurofilament alterations have been reported by others. In this regard, lack of insulin brain stimulation induces JNK hyperphosphorylation followed by hyperphosphorylation of tau and neurofilament damage showing as ultrastructural bunches [40]. In addition, neurofilaments can be GLcNAcylation/phosphorylation during diabetes leading to dysfunctional proteins [41]. 

As expected consequence of brain damage, necrosis, and/or apoptosis could take place during diabetic encephalopathy. In this study apoptosis was found increased in the different regions of the brain but did not reach significant statistic values, suggesting that diabetic encephalopathy could be related to necrosis and/or to dysfunctional disorders. Hyperglycemia observed in this study could induce cellular death by enhancing tissue acidosis [17].

Structural and ultrastructural brain changes were accompanied by depression in diabetic animals. Our results showed that diabetic animals had basal depressive behavior as shown by decreased struggle time and ambulatory activity. These data suggest that depression is involved in experimental diabetes and agree with the clinical depression observed in diabetic humans [42]. Cerebral cortex, cerebellum, and hypothalamus have been shown to be involved in depression. Cerebral metabolism abnormalities have been reported in patients with major and bipolar depression [43, 44]. Studies based on the hypothesis that metabolism impairment might be involved in the pathophysiology of depression, have shown that mitochondrial complexes I, III, and IV were inhibited only in cerebral cortex and cerebellum in chronic stress-induced depression in rats [45]. In addition, mitochondrial citrate synthase used as a marker of mitochondrial function has been reported decreased in depressed patients [46]. Thus, the mitochondrial ultrastructural alterations observed in cerebellum and cerebral cortex in this study could be involved in those functional alterations. In addition to functional impairment, cerebral cellular damage has been reported in experimental depression. In this regard, chronic stress/depression increases the vulnerability of neurons in the rat cerebral cortex by increasing caspase-3 positive neurons, suggesting apoptosis [47], a phenomenon observed in this study. The potential role of cerebellum in the pathophysiology of psychiatric disorders has also been reported. Structural and functional cerebellar abnormalities have been related to bipolar disorder, major depressive disorder and other psychiatric alterations [48, 49]. Structural and ultrastructural alterations in hypothalamus observed in this study could be related to depressive mood in rats. In this regard, dysregulation of the hypothalamus-pituitary-adrenal (HPA) axis is frequently observed in human and experimental depression [50, 51]. 

In conclusion, our results support previous findings and add new information in the structural and ultrastructural alterations in the diabetic rat brain. Although it is difficult to extrapolate our findings to the human condition, the morphological alterations observed in this report may be one mechanism in the pathophysiology of depressive disorders. 

## Figures and Tables

**Table 1:** Control and diabetes parameters.

Parameters	Control (*n* = 10)	Diabetes (*n* = 8)
Blood glucose (mg/dL)	127.5 ± 21.8	306.4 ± 84.66^a^
Weight gain (g)	287.9 ± 39.2	179.4 ± 82^a^
Urine glucose	−	+
Urine ketoacids	−	−

^a^ *P* < .05 versus control.

**Table 2:** Percentages of mitochondrial alterations in diabetes.

	Control	Diabetes	*P* values
**Cerebrum **			
*Cellular body*			
VM^a^	2.1 ± 2.4	25.7 ± 9.81	*P* = .01
SM^b^	4.73 ± 1.97	29.4 ± 8.9	*P* = .01
Total	6.79 ± 1.8	55.13 ± 5.5	*P* = .005
*Neuropil*			
VM	2.0 ± 2.3	27.9 ± 4.11	*P* = .0004
SM	4.60 ± 3.3	14.65 ± 4.24	*P* < .04
Total	6.6 ± 2.9	36.16 ± 15.78	*P* < .04

**Cerebellum**			
*Cellular body*			
VM	5.13 ± 3.5	28.8 ± 2.2	*P* < .006
SM	4.45 ± 1.8	20.5 ± 4.6	*P* < .006
Total	9.55 ± 4.97	49.32 ± 5.79	*P* < .006
*Neuropil*			
VM	3.3 ± 2.8	34.3 ± 1.74	*P* < .002
SM	1.68 ± 1.73	13.16 ± 7.66	*P* < .002
Total	4.95 ± 3.6	47.42 ± 8.8	*P* < .002

**Hypothalamus**			
*Cellular body*			
VM	3.85 ± 1.06	29.35 ± 12.31	*P* = .02
SM	3.83 ± 3.06	21.28 ± 5.07	*P* < .005
Total	7.65 ± 4.06	50.61 ± 8.24	*P* < .005
*Neuropil*			
VM	6.03 ± 2.25	25.63 ± 8.67	*P* < .04
SM	1.9 ± 0.74	9.0 ± 3.97	*P* < .04
Total	7.93 ± 2.02	34.64 ± 11.84	*P* < .04

^a^VM: vacuolated mitochondria; ^b^SM: swollen mitochondria.

**Table 3:** Percentages of myelin alterations in diabetes.

Cerebral cortex	Control	Diabetes	*P* values
Alteration type			
I	4.33 ± 3.10	9.56 ± 2.98;	NS
II	2.17 ± 1.57	4.72 ± 3.36	NS
III	0	1.43 ± 1.2	
Total	5.85 ± 6.57	15.74 ± 5.51	NS

**Cerebellum**			
Alteration type			
I	6.17 ± 4.48	13.68 ± 11.5	NS
II	3.84 ± 3.37	33.12 ± 10.32	*P* = .001
III	1.01 ± 1.36	35.51 ± 20.28	*P* = .01
Total	11.02 ± 4.58	79.85 ± 17.18	*P* < .0002

**Hypothalamus**			
Alteration type			
I	8.06 ± 2.07	19.98 ± 5.7	*P* < .0001
II	2.68 ± 2.28	28.34 ± 3.28	*P* < .0001
III	0	11.51 ± 4.21	
Total	10.75 ± 4.19	59.83 ± 9.64	*P* < .0001

**Table 4:** Forced swimming test and motor activity in controls and diabetic rats.

Parameter	Control	Diabetes
Struggle time (seconds)	27.36 ± 11.46	16.2 ± 6.10^a^
Ambulatory activity (pulses)	238.4 ± 160.5	113.25 ± 36.25^a^
Number of movements (pulses)	59.4 ± 23.7	35.0 ± 15.57^a^

^a^ *P* < .05 versus control.
